# Recent advances in the prevention and treatment of respiratory syncytial virus disease

**DOI:** 10.1099/jgv.0.002095

**Published:** 2025-04-09

**Authors:** Alexandra Sanchez-Martinez, Tom Moore, Telma Sancheira Freitas, Tami R. Benzaken, Shaun O’Hagan, Emma Millar, Helen E. Groves, Simon B. Drysdale, Lindsay Broadbent

**Affiliations:** 1 School of Biosciences, Faculty of Health and Medical Sciences, University of Surrey, Guildford, UK; 2 Medical Radiation Physics Group, The National Physical Laboratory, Teddington, UK; 3 School of Mathematics and Physics, Faculty of Engineering and Physical Sciences, University of Surrey, Guildford, UK; 4 Immunisations and Vaccine Preventable Disease Division, United Kingdom Health Security Agency, London, UK; 5 Department of Paediatric Infectious Diseases, Royal Belfast Hospital for Sick Children, 274 Grosvenor Rd, Belfast, BT12 6BA, UK; 6 Acute Paediatric Medical Services, Antrim Area Hospital, Bush Road, Antrim, BT41 2RL, UK; 7 Wellcome-Wolfson Institute for Experimental Medicine, Queen’s University Belfast, 97 Lisburn Road, Belfast, BT9 7BL, UK; 8 Oxford Vaccine Group, Department of Paediatrics, University of Oxford, Oxford, UK; 9 The NIHR Oxford Biomedical Research Centre, Oxford, UK

**Keywords:** antivirals, mAbs, respiratory syncytial virus, vaccines

## Abstract

Respiratory syncytial virus (RSV) is associated with considerable healthcare burden; as such, prevention and treatment of RSV have long been considered a priority. Historic failures in RSV vaccine development had slowed the research field. However, the discovery of the conformational change in the RSV fusion protein (F) has led to considerable advancements in the field. The RSV pharmaceutical landscape has drastically changed in recent years with successful trials of both vaccines and second-generation mAbs leading to licensing and roll-out of these agents in several countries. RSV preventative and therapeutic measures will likely have a significant impact on RSV-related morbidity and mortality. However, there are still gaps in the protection that these immunizations offer that should be addressed. Many unanswered questions about RSV infection dynamics and subsequent disease should be a focus of ongoing research. This review discusses the currently licensed RSV pharmaceuticals and others that have recently progressed to clinical trials.

## Introduction

Respiratory syncytial virus (RSV) is associated with considerable healthcare burden; as such, prevention and treatment of RSV have long been considered a priority. Historic failures in RSV vaccine development had slowed the research field. However, the discovery of the conformational change in the RSV fusion protein (F) has led to considerable advancements in the field. The RSV pharmaceutical landscape has drastically changed in recent years with successful trials of both vaccines and second-generation mAbs leading to licensing and roll-out of these agents in several countries. RSV preventative and therapeutic measures will likely have a significant impact on RSV-related morbidity and mortality. However, there are still gaps in the protection that these immunizations offer that should be addressed. Many unanswered questions about RSV infection dynamics and subsequent disease should be a focus of ongoing research.

This review discusses the currently licensed RSV pharmaceuticals and others that have recently progressed to clinical trials.

## Respiratory syncytial virus

RSV is a single-stranded, negative-sense RNA virus that belongs to the genus *Orthopneumovirus*, in the *Pneumoviridae* family. The non-segmented genome consists of ten genes that encode 11 proteins [1]. There are three surface glycoproteins: the attachment protein (G), the small hydrophobic protein (SH) and the fusion protein (F) [2]. Two RSV subgroups exist, RSV-A and RSV-B, which are distinguished by genetic and antigenic differences in the G gene and protein, whereas the F protein is highly conserved between subgroups and strains of RSV. The F protein of RSV, which is required for entry into host cells, undergoes a conformational shift from a highly unstable prefusion F (pre-F) trimer confirmation to a much more stable postfusion F (post-F) conformation [3]. This discovery redefined the research field, revealing information about antibody binding and neutralization that has been the cornerstone of many recent developments in RSV pharmaceuticals. The immunogenicity and highly conserved nature of the F protein make it the ideal target for vaccine design.

## RSV epidemiology

RSV is the most common cause of lower respiratory tract infections (LRTIs) in infants [4] and a significant cause of severe LRTI in older adults. RSV is highly contagious, with a typical incubation period of 3–5 days, spreading person-to-person via respiratory droplet transmission or direct contact with contaminated surfaces [5]. Infection with RSV can present with symptoms ranging from mild upper respiratory tract infections to severe LRTI, with particular groups known to be at higher risk of severe disease. These high-risk groups include infants born prematurely and those with chronic lung disease, congenital heart disease and trisomy 21 [6]. Additionally, immunocompromised adults and those over 65 years old, particularly with chronic comorbidities such as chronic obstructive pulmonary disease (COPD), are at significantly increased risk of severe RSV-related LRTI [7].

In temperate regions, RSV demonstrates seasonality, resulting in annual epidemics each winter [8]. Globally, RSV is responsible for over 3 million hospital admissions and more than 100,000 deaths in under 5 year olds each year, with most of the disease burden occurring in children aged less than 1 year old [9, 10]. In England and Wales, RSV-attributable respiratory disease among children accounts for over 29,000 hospitalizations each year, and RSV infection represents 79% of all hospitalizations for bronchiolitis in children less than 6 months old [11]. This results in a considerable cost burden, with an estimated £80 million in annual healthcare costs and productivity losses to the UK economy from RSV infections in children under the age of 5 years. Under-detection of RSV in both paediatric and older adult populations is likely, resulting in challenges in accurately determining RSV-related morbidity and mortality. However, within this population, RSV is known to result in a considerable healthcare burden, with a recent review estimating RSV incidence and hospitalization rates in industrialized countries of ~600 and 150 cases per 100,000 person-years, respectively, with mortality rates above 9% in developing countries [12].

The impact of early-life respiratory infection on lifelong lung health should also be considered when discussing RSV as a pathogen of importance. LRTI during early childhood correlates with an increased risk of premature death due to respiratory disease. Severity of infection and age of the first LRTI further correlate with this risk [13]. Early-life RSV infection has also been linked to recurrent wheeze (RW) and childhood asthma, although causality is debated [14].

## RSV vaccine history and the discovery of pre-F protein

Since the discovery of RSV as a causative agent of disease in humans and its capacity to severely affect infants nearly 70 years ago, significant efforts have been made to develop prophylactic measures through both active and passive immunization. The first RSV vaccine candidate, a formalin-inactivated (FI) RSV vaccine, was developed in 1966 [15]. During clinical trials, the FI-RSV vaccine resulted in enhanced respiratory disease (ERD) when the RSV-naïve vaccinated children were subsequently infected with RSV in the community [16]. This resulted in the death of two trial participants [16]. The underlying cause of ERD following FI-RSV vaccination was not evident until 2016. Killikelly *et al*. demonstrated that FI-RSV predominantly presents post-F conformation on the virion surface, whereas infectious RSV presents pre-F and post-F conformations [17]. This contradicted the assumption that the formalin treatment of RSV would only produce an inactivated virus; instead, the FI-RSV vaccine changed the virus antigenicity, inducing elevated titres of binding antibodies with low neutralizing activity.

The pre-F conformation induces a much more protective immune response than the post-F conformation [3]. Pre-F contains the most neutralizing sites (Ø and V) on its surface, whereas sites I, II and IV are conserved between pre- and post-F conformations [18]. Pre-F is unstable and is liable to flip to the more stable post-F. Advances in structure-based protein design led to stabilizing the F protein in the pre-F conformation, allowing the antigenic site Ø to be maintained under extreme environmental conditions [18]. McLellan *et al*. demonstrated that an engineered pre-F protein variant with site Ø-stabilized (DS-CaV1) could induce about 80-fold higher neutralization capacity during *in vivo* preclinical testing than post-F [19].

The discovery of the relevance of pre-F protein in generating a protective immune response, combined with technological advances that allowed the stabilization of the pre-F conformation, has paved the way for the rapid development of several promising vaccine candidates over the last decade. Most RSV vaccines that are either licensed or in development use the stabilized pre-F protein conformation as the basis for their vaccine antigen design.

## Advances in RSV vaccine strategies

**Fig. 1.**
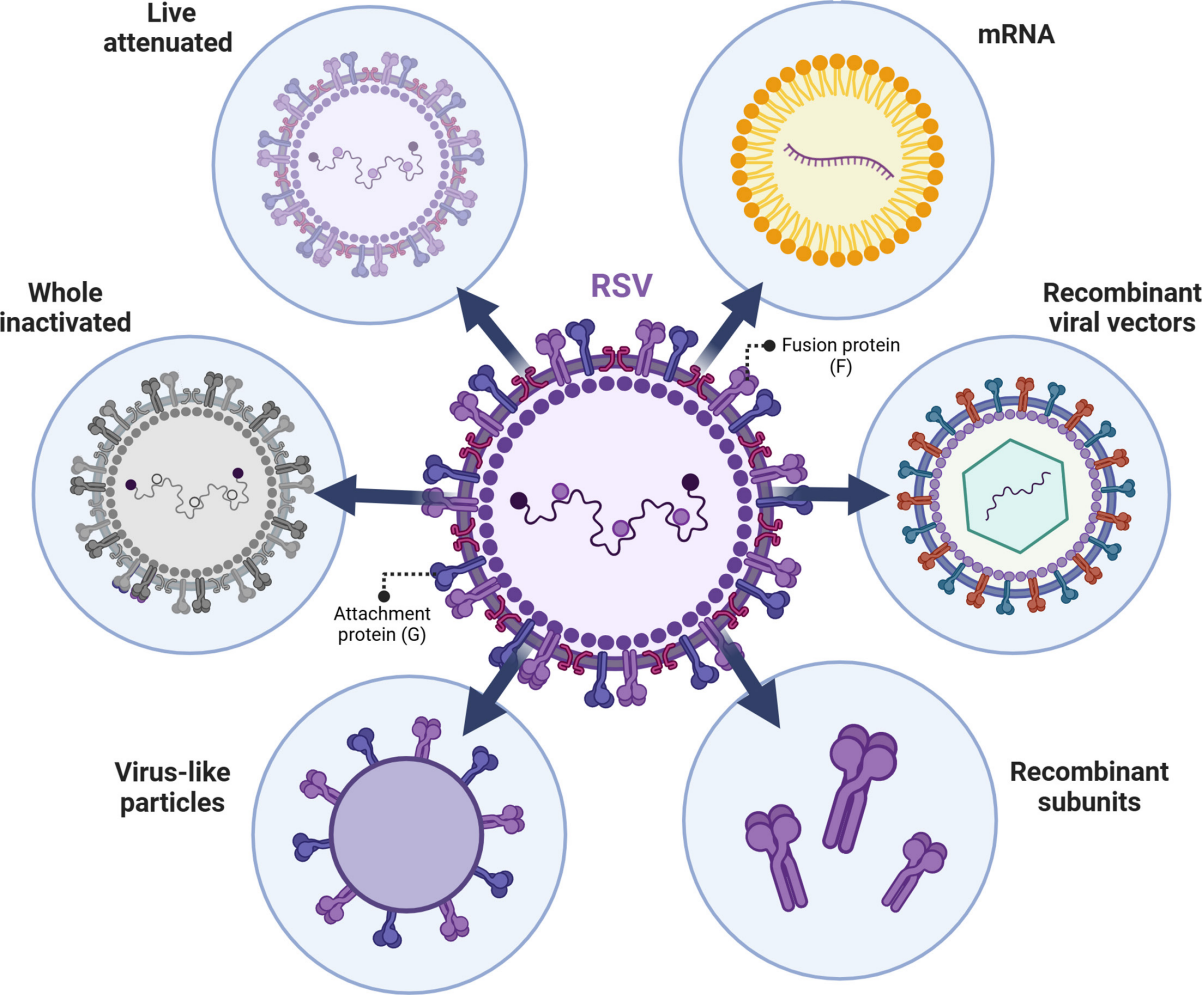
Recent approaches for RSV vaccine development. Recent RSV vaccine strategies include LAVs, whole-inactivated viruses, VLPs, subunit proteins, recombinant viral vectors and mRNA vaccines. The viral F and G proteins are the major targets for RSV vaccine antigen design.

Several differing approaches to vaccine development have been adopted (Fig. 1), including live-attenuated vaccines (LAVs), whole-inactivated viruses, virus-like particles (VLPs), subunit proteins, recombinant viral vectors and mRNA vaccines [20]. Vaccine candidates progressing to clinical trials from phase 2 are listed in Table 1.

**Table 1.** RSV vaccine clinical trials for vulnerable groups. Clinical trials listed as phase 2 and phase 3 in ClinicalTrials.gov as of 05 November 2024. ‘RSV vaccine’ was used as a search term. Clinical trials with infants/toddlers, pregnant women, adults at higher risk and older adults as participants were included

Vaccine	Type of vaccine	Description	Route	Target population/trial	Phase/status	Year	Results
mRNA-1345 (mRESVIA, Moderna)	mRNA	Lipid nanoparticle-encapsulated mRNA-based vaccine encoding the membrane-anchored RSV F glycoprotein, derived from an RSV-A strain and stabilized in the preF conformation.	i.m.	Older adults (ConquerRSV)	Phase 3/authorized vaccine (NCT05127434)	2024	mRESVIA achieved the primary efficacy endpoint of preventing RSV-LRTI with at least two/three signs or symptoms [86].
				Pregnant women	Phase 2 (NCT06143046)	2023–2026	Not available
RSVPreF3 (Arexvy, GSK)	Protein subunit	Stabilized pre-F glycoprotein adjuvanted with AS01E.	i.m.	Older adults (AReSVi-006)	Phase 3/authorized vaccine (NCT04886596)	2023	RSVPreF3 had an acceptable safety profile and prevented RSV-ARI and LRTI and severe RSV-LRTI disease in older adults, regardless of the RSV subtype [50].
				Pregnant women (GRACE)	Phase 3 (NCT04605159)	2023	Trial enrolment was stopped early because of safety concerns. The risks of RSV-LRTI among infants were lower with the maternal RSV vaccine than with placebo, but the risk of preterm birth was higher [68].
				Healthy non-prepregnant girls (9–17 years) and non-pregnant adult women (18–49 years)	Phase 3(EUCTR2021-004003-41-ES)	2024	Trial enrolment stopped early, in March 2024, following the recommendation from the Independent Data Monitoring Committee. The eight enrolled patients were monitored until study completion.
RSVpreF (Abrysvo, Pfizer)	Protein subunit	Bivalent vaccine containing the stabilized preF glycoproteins from the two major cocirculating antigenic subtypes (RSV-A and RSV-B).	i.m.	Adults at higher risk (MONeT)	Phase 3/authorized vaccine (NCT05842967)	2024	Participants demonstrated RSV-A and RSV-B neutralizing responses non-inferior to those observed in the Phase III RENOIR study. No safety concerns were found.
				Pregnant women (MATISSE)	Phase 3/authorized vaccine (NCT04424316)	2023	RSVpreF administered during pregnancy was effective against severe RSV-LRTI in infants without safety concerns [64].
				Older adults (RENOIR)	Phase 3/authorized vaccine (NCT05035212)	2023	RSVpreF prevented RSV-LRTI and RSV-ARD in older adults, without safety concerns [53].
SP0125, ΔNS2/Δ1313/I1314L (Sanofi)	LAV	ΔNS2/Δ1313/I1314L contains the deletion of the viral non-structural protein 2 (NS2), an antagonist of host interferon and apoptosis responses and a single-codon deletion in the ORF encoding the polymerase protein L resulting in an attenuated and temperature-sensitive vaccine. PMID: 35732186	i.n.	Infants and toddlers (6–22 months) (PEARL)	Phase 3 (NCT06252285)	2024–2027	Results are not available yet.
MVA-BN-RSV (Bavarian Nordic)	Attenuated viral vector	Modified vaccinia Ankara (MVA) vector platform encoding five RSV antigens: F, two surface proteins G of different subtypes (subtype A and subtype B) and two internal proteins: nucleoprotein (N) and transcription elongation factor (M2-1).	i.m.	Older adults (VANIR)	Phase 3 (NCT05238025)	2023	MVA-BN-RSV failed to meet the co-primary endpoint of severe LRTI based on at least three predefined symptoms, demonstrating a 42.9% efficacy. However, MVA-BN-RSV had 59% efficacy in preventing at least two predefined LRTI symptoms [120].
Ad26.RSV.preF (Janssen)	Attenuated viral vector	Adenovirus serotype 26 (Ad26) vector-based vaccine that encodes the stabilized pre-F RSV.	i.m.	Older adults (EVERGREEN)	Phase 3 (NCT04908683)	2023	EVERGREEN was initiated based on positive results from the Phase 2b CYPRESS trial, demonstrating 80% efficacy against confirmed RSV-LRTI and tolerability. However, in April 2023, the trial was discontinued as part of a strategic decision by the pharmaceutical company Johnson and Johnson [53].
				Adults and toddlers	Phase 1/2 (NCT03303625)	2020	Ad26.RSV.preF had an acceptable safety profile, was well tolerated and elicited humoral and cellular immune responses in adults and RSV-seropositive children (12–24 months). Fewer RSV infections were observed in Ad26.RSV.preF recipients compared with placebo recipients [121].
ResVax (Novavax)	Nanoparticle	RSV F protein recombinant nanoparticle vaccine formulated with aluminium phosphate as an adjuvant.	i.m.	Pregnant women (Prepare)	Phase 3 (NCT02624947)	2019	ResVax did not meet the primary endpoint of preventing MA RSV-LRTI through 90 days of life [31].
IVX-A12 (Icosavax, Inc/AstraZeneca)	VLP	IVX-A12 is composed of two VLPs that incorporate the stabilized pre-F proteins from RSV (IVX-121) and HMPV (IVX-241) combined with MF59 adjuvant.	i.m.	Older adults	Phase 2 (NCT05903183)	2023–2025	IVX-A12 promoted the increase of RSV- and HMPV-specific antibodies. IVX-A12 was generally well-tolerated, with a safety profile similar to that seen in the Phase 1 trial [122].
BARS13 (Advaccine Biopharmaceuticals Suzhou)	Protein subunit	Recombinant RSV G protein combined with cyclosporine A (CsA).	i.m.	Older adults	Phase 2 (NCT04681833)	2024	The study demonstrated the safety and tolerability across different dose groups. Levels of antibodies exhibited a dose- and frequency-dependent response [35].
ChAd155-RSV (GSK3389245A) (GSK)	Attenuated viral vector	Chimpanzee-derived Adenovector expressing three RSV proteins (F, N and M2-1).	i.m.	RSV-seropositive toddlers (12–23 months) (PED-002)	Phase 1/2 (NCT02927873)	2020	Three ChAd155-RSV doses were well-tolerated. A dose-dependent immune response was observed after dose 1, with no observed booster effect after dose 2 [123].
RSVΔNS2/Δ1313/I1314L, RSV 6120/ΔNS2/1030s, RSV 276(NIAID)	LAV	RSV ΔNS2/Δ1313/I1314L is attenuated by the NS2 gene deletion and a temperature-sensitivity mutation in the polymerase gene.RSV/6120/ΔNS2/1030s is a cDNA-derived live vaccine attenuated by (1) deletion of the interferon antagonist NS2 gene and (2) the genetically stabilized 1030s missense polymerase mutation in the polymerase.RSV 276 is attenuated by M2-2 deletion.	i.n.	RSV-seronegative infants (6–24 months)	Phase 1/2(NCT03916185)	2024	Results are not available yet.

i.m., Intramuscular injection; i.n., Intranasal injection.

RSV LAVs are promising candidates for the paediatric population. These vaccines have demonstrated the ability to induce innate and adaptive immune responses, both systemically and locally, within the respiratory tract. As a result, this vaccine strategy is considered ideal for intranasal administration. Conventional LAVs involve the adaptation of virulent viruses to novel hosts, cell cultures or suboptimal environments, reducing their virulence but maintaining their ability to mimic natural infection and stimulating protective immune responses [21]. The most advanced RSV LAV candidate in phase 3 clinical trials is SP0125 (Sanofi), or RSV/ΔNS2/Δ1313/I1314L. SP0125 has been shown to elicit protective humoral responses in children with good tolerability [22]. SP0125 contains a 523 nt deletion of the viral non-structural protein 2 (NS2), an antagonist of host interferon and apoptosis responses. It includes a single-codon deletion (Δ1313) in the ORF encoding the polymerase protein L, combined with the missense mutation I1314L that increases the phenotypic stability of Δ1313 [23]. The resulting candidate is attenuated and moderately temperature-sensitive. A promising mucosal bivalent LAV candidate to prevent pneumovirus-induced diseases is the mucosal bivalent LAV candidate, known as Metavac-RSV [24]. In this candidate, the RSV F gene was inserted into an Human metapneumovirus (HMPV)-based LAV (Metavac), exposing both RSV and HMPV F proteins at the virion surface [24].

Novel, non-formalin inactivation strategies have been explored to develop whole-inactivated vaccine candidates for RSV. Among these, ionizing radiation shows promise as a method of RSV inactivation. Low-energy electron irradiation (LEEI) can inactivate RSV with 70% conservation of viral antigens [25]. Intramuscular administration in mice results in a strong immune response with a high titre of neutralizing antibodies. Ongoing research is investigating the efficacy of intranasal administration of LEEI-inactivated RSV packaged in liposomes [26]. Gamma radiation (photons) has also been used to inactivate RSV [27]. Gamma radiation results in relatively low levels of protein carbonylation, which was previously thought to cause the conformational change in the F protein [28]. However, gamma-inactivated RSV induced ERD in mice due to high levels of post-F, and the conformational change resulted from oxidative stress, not carbonylation as previously thought. The pre-F conformation can be preserved using reactive oxygen species scavengers [29]. Research in this area is ongoing.

VLPs are non-traditional vaccine strategies for RSV vaccine design. VLPs mimic the form and size of a viral particle but lack the genetic material and can be engineered to display an antigen per particle of multivalent combinations [30]. X-A12 (Icosavax, Inc/AstraZeneca) is a formulation comprised of two VLPs, IVX-121 and IVX-241, containing the RSV and HMPV pre-F proteins, respectively [31]. X-A12 has shown robust humoral immune responses across RSV and HMPV antigens and evidenced an excellent safety profile, progressing to phase 2 clinical trials (NCT05903183). Recent VLP platforms include the combination of multiple proteins on the surface of the VLPs, showing higher immunogenicity and protection than soluble proteins or monovalent VLPs. HIV Gag-derived VLPs that express RSV and HMPV F proteins show better immunogenicity and neutralizing response than soluble F proteins [32]. Co-expressing pre-F protein with G protein on VLPs has been shown to elicit higher protection and a more robust Th1-type immune response than single antigen-expressing VLPs [33,34].

Recombinant protein subunit vaccines include the first RSV vaccines authorized for human use, RSVPreF3 (Arexvy, GSK) and RSVpreF (Abrysvo, Pfizer). These subunit vaccines contain the stabilized RSV pre-F protein; antigen design, clinical trials and current use are described in the following section: Authorized RSV vaccines . BARS13 (Advaccine Biopharmaceuticals Suzhou) is another protein subunit candidate, consisting of the RSV G protein, which is currently in phase 2 clinical trials (NCT04681833) [35]. Innovative approaches include the development of more stable pre-F protein conformation using dityrosine crosslinks, which significantly induce greater immunogenicity than DS-CaV1 [36]. Recombinant protein-based vaccines also include nanoparticle (NP) vaccines, which incorporate a given target antigen and are assembled through various chemical and physical processes into a nanosized vehicle for delivery [37,38]. ResVax (Novavax) has been the furthest polymer-based NP vaccine candidate in progress. ResVax consists of RSV pre-F protein stabilized around a core of polysorbate 80 adjuvanted with aluminium phosphate. However, the vaccine did not reach the primary endpoint in phase 3 clinical trials evaluated in pregnant women [31]. In pre-clinical studies, additional polymer-based vaccines have been evaluated, including polyanhydride NPs, synthetic thermoresponsive polymer chains and poly(d,l-lactide-*co*-glycolide) [38,39].

Recombinant attenuated viral vector strategies have demonstrated significant potential for developing effective RSV vaccines by utilizing the immunogenicity of viral vectors to deliver RSV antigens. Phase 3 clinical trials have included MVA (modified vaccinia Ankara)-BN-RSV (Bavarian Nordic) and Ad26.RSV.preF (Janssen); however, these attenuated viral vectors did not progress to marketing authorization. MVA-BN-RSV is a poxvirus-vectored vaccine that uses an MVA virus encoding five RSV antigens: F, G from subtypes A and B, N and M2-1. MVA-BN-RSV did not reach its primary clinical trial endpoint of prevention of RSV-LRTI (≥3 symptoms) in older adults, showing only 42.9% efficacy [40]. Ad26.RSV.preF is an adenovirus serotype 26 (Ad26) vector-based vaccine that delivers the stabilized pre-F protein. Although the heterologous prime-boost administration of Ad26.RSV.preF combined with pre-F demonstrated 80% efficacy in a phase 2b clinical trial, subsequent trials were discontinued in response to strategic marketing considerations [41]. Novel attenuated viral vector strategies in pre-clinical testing include BLB201, a live viral-vectored candidate RSV vaccine based on a parainfluenza virus 5 encoding the RSV F antigen administered intranasally [42].

Advances in mRNA vaccine technology have progressed rapidly. The degradation in mRNA was overcome by synthesizing it using modified uridine [43]. The mRNA vaccine mRESVIA (Moderna) encodes the stabilized RSV pre-F protein and has been authorized for human use as described in section 6. Whilst three mRNA vaccines have been approved for COVID-19, mRESVIA is the first mRNA-authorized vaccine to target a different pathogen. Recent pre-clinical efforts have developed bivalent combinations, including RSV and SARS-CoV-2 antigens [44].

## Authorized RSV vaccines

As previously discussed, although vaccine development for RSV began in the 1960s, it was not until 2023 that the first RSV vaccines became licensed and available for human use. In May 2023, the world’s first RSV vaccine, Arexvy (GSK), was approved by the United States Food and Drug Administration (US FDA) to prevent RSV-LRTI in adults aged 60 years and older [45]. Shortly thereafter, Arexvy also gained approval from the European Medicines Agency (EMA) and the Medicines and Healthcare Products Regulatory Agency (MHRA) in the UK [46]. Abrysvo (Pfizer) was the second RSV vaccine candidate to receive US FDA approval in May 2023 [47] and was also licensed in Europe and the UK during the same year [48]. Abrysvo was initially authorized to prevent RSV-LRTI in older adults and was the first RSV vaccine to receive approval for passive immunization of infants through maternal administration during pregnancy and for adults between 18 and 59 at higher risk of RSV [46]. In May 2024, the first RSV mRNA vaccine, mRESVIA (Moderna), received marketing authorization from the EMA and the US FDA to protect adults aged 60 years and older [49]. Whilst all approved RSV vaccines utilize the pre-F protein as the primary antigen, significant distinctions exist among them in terms of design, delivery and efficacy.

Arexvy is a subunit protein monovalent vaccine containing a stabilized pre-F protein (based on a laboratory-adapted RSV-A strain) adjuvanted with AS01E [50]. The antigen used in Arexvy is derived from the pre-F-stabilized protein DS-Cav1. The conformation of DS-Cav1 was initially designed using the RSV pre-F conformation when bound to the D25 mAb [19]. Using the D25-bound pre-F structure as a template for DS-Cav1 design was crucial, as D25 specifically targets the pre-F conformation and helps stabilize both overall and local RSV F conformations [19]. DS-Cav1 was also engineered to retain the metastable antigenic site Ø when exposed to extreme environmental conditions (temperature, pH, osmolality and freeze-thaw) [19]. The pre-F-stabilized conformation contains two intra-chain cysteine modifications, S155C-S290C (double mutant DS); this creates a covalent link between two cysteine residues, helping to lock the protein in its prefusion conformation [19]. The hydrophobic substitutions or cavity-filling mutations S190F and V207L (cav1) were also included [19]. These cavity-filling mutations are a type of genetic modification in proteins, where a smaller aa is replaced by a larger one to fill an internal cavity in the protein structure, enhancing protein stability by strengthening hydrophobic interactions in the protein core [51]. DS-Cav1 is also linked to a T4 fibritin trimerization domain (foldon) at its C-terminal end [19]. This foldon comprises 30 aa that assemble into a stable *β*-propeller structure with a hydrophobic core, which assists in retaining the trimeric state of the antigen [52].

Abrysvo is a subunit-based vaccine containing the stabilized pre-F proteins in equal amounts of the two major cocirculating antigenic subtypes (RSV-A and RSV-B) [53]. Abrysvo is a non-adjuvanted formulation reconstituted with sterile water [53]. The bivalent RSV pre-F vaccine was obtained after screening nearly 400 engineered F proteins for stability under multiple stress conditions (heat, storage and urea) and binding to conformation-specific mAbs [54]. All proteins included the fibritin foldon trimerization domains at their C-terminal ends. The top candidate was construct 847, which carries stabilizing disulphide bond mutations (T103C-I148C), a cavity-filling mutation (S190I) and a charge neutralization mutation (D486S) [54]. Replacement of a charged aa with a neutral one helps to reduce ionic repulsion or enhance ionic attraction between residues proximate to each other at the trimer interface in the prefusion conformation [55]. The stabilizing mutations of construct 847 were introduced onto the F protein backbones of RSV-A and RSV-B strains [54]. Construct 847 elicited higher RSV-neutralizing antibodies than DS-Cav1 during immunogenicity studies in cotton rats. Although the RSVF glycoprotein is conserved across RSV strains, structural analysis has indicated the sequence differences between RSV-A and RSV-B clusters in the antigenic site Ø [54]. Thus, Abrysvo potentially offers broader protection against RSV, regardless of the virus subgroup.

The vaccine mRESVIA also consists of an mRNA sequence encoding a stabilized RSV pre-F glycoprotein encapsulated in lipid NPs (LNPs). The development of mRESVIA was based on the initial evaluation of immunogenicity, protection and safety in rodent models of mRNA constructs with the sequences of RSV F proteins. The pharmaceutical Merck developed these initial screenings, and the studies included mutations designed to stabilize RSV F in its pre-F conformation (DS-Cav1), including both full-length (membrane-associated, mDS-Cav1) and ectodomain forms (secreted, sDS-Cav1) and monomeric, trimeric and non-stabilized forms of the antigen [56]. The mRNA vaccine candidates expressing the stabilizing mutations had more antibodies targeting the antigenic site ∅ than native forms. However, stabilized or native forms of RSV pre-F protein elicit robust neutralizing humoral responses in both mice and cotton rats, similar to levels observed with a comparable dose of adjuvanted DS-Cav1 [56]. However, independent of their conformations, the mRNA vaccine candidates elicited higher T-cell responses in mice [56]. This enhanced T-cell response is a key advantage of the mRNA vaccine technology compared to protein subunit vaccines. Consequently, the vaccine candidate mRNA-1777, encoding the full-length RSV F protein stabilized in the pre-F conformation (mDS-Cav1) formulated with MC3 (also known as DLin-MC3-DMA) LNP delivery system, was evaluated in a phase 1 clinical trial [57]. mRNA-1777 was well tolerated when administered to young and older adults and elicited an increase in RSV humoral and cell-mediated immune responses [57].

A next-generation mRNA-based RSV F vaccine, mRNA-1172, was designed to improve its predecessor, mRNA-1777. mRNA-1172 retained mDS-Cav1 mutations included in mRNA-1777 but also included interprotomer disulphides, which are additional disulphide bonds introduced between protomers of the RSV F protein trimer to enhance stability and the fusion of the F1 and F2 subunits to create a single-chain version, further improving stability [58]. mRNA-1172 showed enhanced immunogenicity compared to mRNA-1777. Both vaccines were formulated with L-608 LNP instead of MC3 [59]. mRNA-1345 (mRESVIA) included codon redesign and deletion of the cytoplasmic tail (ΔCT) on the mRNA-1172 construct for higher protein expression *in vitro* [60]. The mRNA-1345 candidate induced a Th1-type immune response, neutralizing humoral responses with IgG titres targeting the antigenic site Ø in pre-clinical testing [60]. Importantly, in addition to the modifications on the mRNA constructs, the clinical studies of mRNA-1177, mRNA-1172 and mRNA-1345 utilized different LNPs. mRNA-1345 uses the same LNPs as the Moderna COVID-19 vaccines, the ionizable lipid, SM-102 [61].

RSV vaccine development strategies target key populations that are particularly more vulnerable to severe RSV-related disease, including maternal and paediatric (infants and toddlers) populations, older adults and individuals in increased-risk populations. The current efficacy and administration of the approved RSV vaccines across these vulnerable populations are delineated as follows.

### Maternal RSV vaccination

Maternal immunization provides passive immunity to newborns by transferring maternal antibodies across the placenta. In contrast to infants, most adults have been naturally infected with RSV and have detectable neutralizing antibody titres. Immunization of pregnant women could reduce the risk of ERD and passively protect infants before the peak incidence of RSV disease, which typically occurs at 6 weeks of age [62]. In addition, palivizumab, a mAb targeting RSV F, in RSV-naïve infants suggests that neutralizing antibodies are sufficient to protect against severe RSV-LRTI in this population [63].

Abrysvo is the only currently approved vaccine for maternal immunization [64]. In a phase 2b clinical trial, Abrysvo demonstrated its effectiveness in eliciting neutralizing antibodies in maternal serum and efficient transplacental transfer of neutralizing antibodies, which were associated with medically attended RSV-associated LRTI (MA RSV-LRTI) in infants [65]. Approval for Abrysvo followed the results of the international phase 3 randomized placebo-controlled MATISSE (Maternal Immunization Study for Safety and Efficacy) trial. Recruiting 7,400 pregnant women at 24–36 weeks of gestational age, the trial was stopped early after meeting the primary endpoint, demonstrating a vaccine efficacy of 81.8% (99.5% CI 40.6–96.3) for the prevention of severe MA RSV-LRTI during the first 90 days of life. Additionally, the study demonstrated a 69.4% vaccine efficacy against RSV-LRTI in infants up to 6 months of life [64]. However, safety concerns in a second RSV vaccine maternal candidate, Arexvy, prompted initial apprehension, with some experts urging for close post-approval monitoring [66]. In February 2023, phase 3 trials of Arexvy were terminated early due to safety concerns regarding a significant increase in preterm births and neonatal deaths in vaccine recipients compared to placebo [67]. Trial data demonstrated preterm birth in 6.8% of the maternal vaccine cohort, versus 4.9% within the placebo cohort, equivalent to approximately one extra preterm delivery for every 54 vaccinated mothers (*P*=0.01) [68]. Furthermore, there were 13 out of 3,494 (0.4%) neonatal deaths within the vaccine cohort compared to 3 out of 1,739 (0.2%) in the placebo group (*P*=0.23) [68]. The increased risk of preterm birth in the vaccine group was primarily seen in low- and middle-income countries (LMICs), with no other safety signals observed among infant or maternal participants. The increase in preterm births remains unexplained [68].

Additional preterm births were also observed in Abrysvo vaccine recipients, when compared with unvaccinated mothers (5.6 % versus 4.7 %); however, this difference was not statistically significant [64]. As such, the US FDA granted approval for maternal administration at 32–36 weeks’ gestation, reducing the potential for early preterm delivery [69]. Within the European Union (EU), Abrysvo received market approval for administration between 24 and 36 weeks’ gestation, in keeping with the gestational age range within the MATISSE trial. Subsequently, a ‘real-world’ retrospective observational cohort study within two New York City hospitals evaluated the association between maternal RSVpreF vaccination and preterm birth. This study of 2,973 patients demonstrated a favourable preterm safety profile, with no significant difference in preterm births after 32 weeks (vaccinated 5.9% versus unvaccinated 6.7%; 95% CI 0.62–1.20) or neonatal outcomes based on vaccination status [70]. However, the study did report a significant increase in rates of hypertensive disorders of pregnancy following RSVpreF vaccination (hazard ratio 1.43, 95% CI 1.16–1.77), and additional investigation into this risk in future studies is necessitated [70].

In addition to the currently licensed maternal RSV vaccine, several maternal vaccines are in development, including two vaccines in phase 1 clinical trials [71,72] and an NP vaccine that was well tolerated in a phase 2 clinical trial, but failed to reach the primary endpoint of preventing medically significant RSV-LTRI through 90 days of life in a phase 3 clinical trial [31]. Moderna is in the recruitment stages of a phase 2 trial of an mRNA-based investigational vaccine, mRNA-1345, which will be delivered to pregnant women between 28 and 36 weeks’ gestation with the aim of assessing the level of protection in infants within their first few months of life [73].

### Paediatric RSV vaccination

Presently, there are no licensed RSV vaccines for direct administration to the paediatric population. An infant RSV vaccine would ideally be administered within the first month of life, given that the peak incidence of severe disease occurs in infants less than 6 months old. This predisposition to severe disease is related to a dysregulated immune response, with inadequate antibody formation, and impaired T-cell and plasmacytoid dendritic cell function [74,76]. Immune immaturity and the presence of maternal antibodies, both of which may limit vaccine efficacy, pose considerable challenges to vaccine development within this vulnerable group. Additionally, the vaccine-induced ERD significantly set back the development of subunit RSV vaccines, especially amongst RSV-naïve infants.

Sanofi is currently recruiting infants and toddlers 6–22 months old to a multi-centre phase 3 clinical trial evaluating the efficacy, immunogenicity and safety of an LAV candidate (ΔNS2/Δ1313/I1314L) (NCT06252285). With an anticipated enrolment of 5,334 patients, recruitment will be ongoing from May 2024 to November 2028. The National Institute of Allergy and Infectious Disease has three LAV candidates, which were studied in seronegative infants 6–24 months old, in a phase 1/2 trial that was completed in May 2024, with results awaited (NCT03916185). Additionally, two attenuated viral-vector vaccine candidates were well tolerated, with no safety concerns in completed phase 1/2 clinical trials, with further details provided in Table 1.

### Older adults’ vaccination

RSV is a significant contributor to disease burden in older adults. Epidemiological evidence indicates that the annual RSV impact in older adults is ~25–50% of that attributed to influenza type A (subtype H3N2) and is similar to yearly rates of influenza type A (subtype H1N1) and influenza type B (Nuwer 2023). Whilst RSV and seasonal influenza have comparable mortality rates, RSV infection is associated with a higher hospitalization rate and extended hospital stay in older adults [77]. Older adults are highly susceptible to RSV infections and infection-related adverse outcomes due to their immune system’s functional decline (e.g. immunosenescence), particularly the frail elderly and those with underlying conditions, as they are at risk of infection-related pneumonia and death [78]. The effects of ageing on the immune system also contribute to reduced vaccine responsiveness [37]. However, the recently licensed RSV vaccines have met the required efficacy for protection in older adults.

Arexvy was the first vaccine to receive FDA approval for older adults. Arexvy was selected for further clinical development based on its capacity to boost RSVPreF3-specific IgG (geometric mean concentrations were 7.2–12.8 on day 31, 5.5–9.3 on day 91 and 2.6–4.5 at month 14), neutralizing titres [geometric mean litres (GMTs) ranged from 5.6 to 9.9 on day 31, 3.8 to 6.6 on day 91 and 2.7 to 4.4 at month 14 for RSV-A and were similar for RSV-B] and cell-mediated immunity (RSV-specific CD4+ T-cells) responses in older adults [79]. The AReSVi-006 phase 3 trial demonstrated that a single dose of Arexvy had an efficacy of 82.6% against RSV-LRTI, 94.1% against severe RSV-LRTI and 71.7% against RSV-related acute respiratory infection among adults 60 years of age or older during one RSV season [50]. Vaccine efficacy was similar against the RSV-A and B subtypes [50]. The AReSVi-006 trial showed that efficacy over two seasons of one Arexvy dose was 67.2% against RSV-LRTI and 78.8% against severe RSV-LRTI. An additional boosting dose 1 year after dose 1 did not provide additional efficacy benefits in the overall study population [80]. The vaccine efficacy discrepancy between season one and season two has been attributed to a higher RSV circulation and RSV disease in the community in season two than during season one [80]. Limitations of the prelicensure trial include the underrepresentation of groups at increased risk of severe RSV disease, with a small proportion of participants above 80 years of age or older [50].

Abrysvo has been authorized as a single-dose vaccine for all adults above 75 years old and adults above 60 and has been approved for medical use in the USA, the EU, the UK and in multiple countries worldwide [81]. Abrysvo induced RSV-neutralizing titres higher than those associated with protecting high-risk infants by palivizumab (100 µg ml^−1^ serum level) after 1 month of immunization in 18–49-year-old adults [82]. Among older adults aged 65–85 years, Abrysvo increased RSV-A and RSV-B neutralizing GMTs from 15,169–30,071 before vaccination to 14,905–30,071 at 1 month post-vaccination, similar to 18–49-year-old recipients [83]. Significantly, administering RSVpreF with the seasonal inactivated influenza vaccine (SIIV) does not negatively affect the humoral response induced by Abrysvo in older adults [83]. In the RSV Efficacy Study in Older Adults Immunized against RSV Disease (RENOIR) trial, Abrysvo demonstrated a vaccine efficacy of 88.9% against RSV-LRTI with at least three symptoms during the first RSV season, involving participants at least 60 years of age [53]. The vaccine had an efficacy of 84.4% in preventing RSV-LRTI and 81.0% in preventing MA RSV-LRTI during the second RSV season, which indicates durable efficacy after two seasons (RENOIR trial 2024), suggesting that Abrysvo could be administered several months before the start of the RSV season without losing protective effects throughout the season. Subgroup analyses of the primary endpoints according to participant age group (60–69 years, 70–79 years or ≥80 years), RSV-A or RSV-B and risk status indicated similar vaccine efficacy across subgroups [53]. RENOIR excluded immunocompromised persons and those with the most severe comorbidities. Also, vaccine effectiveness (VE) estimates were not available for the oldest age groups. In pre-licensure trials, Guillain–Barré syndrome (GBS) was identified as a potential vaccine safety concern after the administration of the RSV vaccines Arexvy and Abrysvo [84]. Reporting rates of GBS after RSV vaccination were 1.8 and 4.4 reports per million doses of Abrysvo and Arexvy, respectively, which were higher than the estimated expected background rates in a vaccinated population [47]. Nonetheless, the estimated benefits of RSV vaccination outweigh the potential risks, and these vaccines continue to be recommended for older adults.

mRESVIA is also indicated as a single-dose vaccine. It can elicit neutralizing antibodies [geometric mean fold rise (GMFR) ≥2.39 for RSV-A and ≥1.52 for RSV-B], which remain above baseline through 12 months post-dose (GMFR >2.0), demonstrating long-lasting immunity [85]. In addition, the co-administration of mRESVIA with influenza (SIIV4) or COVID-19 (mRNA-1273.214) vaccines is safe and does not have an impact on the immunogenicity of any of the vaccines in older adults. mRESVIA efficacy has been assessed in the ConquerRSV trial, which included participants above 60 years old, randomly selected to receive a single vaccine dose or placebo [86]. mRESVIA efficacy was 82.4% and 68.4% against RSV-LRTI with at least three signs or symptoms of RSV-associated acute respiratory disease (ARD). RSV-ARD was defined as RT-PCR–confirmed RSV infection and at least one new or worsening respiratory symptom for at least 24 h. Vaccine efficacy against RSV-ARD was higher for RSV-A than against RSV-B (78.5% vs 51.7%), and most of the adverse reactions were mild to moderate and transient. A follow-up study reported that a dose of mRESVIA was 50% less effective in preventing RSV-LRTI over 18 months after vaccination in older adults. Hence, one dose of mRESVIA appears less effective over two RSV seasons than one dose of a recombinant RSV vaccine (Arexvy or Abrysvo) [87]. No safety concerns have been identified for mRESVIA.

RSV VE against hospitalization among adults 60 years of age or older without immunocompromising conditions was 80% during the first RSV season [88]. Additional VE studies suggest that VE did not differ among adults aged 60–74 years or 75 years and older [89]. Further analyses are needed to evaluate whether the VE of RSV vaccines is maintained over more than two seasons and if increasing the interval between the vaccine’s first dose and boosting dose may enhance the vaccine efficacy.

### RSV vaccination for adults at increased risk of disease

Adults who are immunocompromised or have an underlying chronic condition, including obesity, diabetes, COPD, heart failure, chronic kidney disease and asthma, are at increased risk of developing RSV-LRTI (NCT05842967). The pivotal phase 3 clinical trial MONeT (RSV IMmunizatiON Study for AdulTs at Higher Risk of Severe Illness) evaluated the safety, tolerability and immunogenicity of Abrysvo in adults 18–59 years of age at risk of developing RSV-LRTI. Substudy A is a double-blinded study that includes participants aged 18–59 with chronic conditions. Substudy B is an open-label study that enrolled immunocompromised adults aged 18 or older, roughly half of whom were 60 or older, receiving two doses of Abrysvo 1 month apart [90]. In substudy A (NCT05842967), Abrysvo demonstrated consistent safety; also, RSV-neutralizing titres in adults 18–59 at high risk of severe RSV disease were non-inferior to older adults [91].

Similarly, preliminary data demonstrated the protective effect of Arexvy in those aged 18–49 at increased risk for RSV-LRTI [92]. In a phase IIIb trial (NCT06389487), a single dose of vaccine elicited a robust immune response with an acceptable safety profile in adults aged 18–49 at increased risk for RSV-LRTI, with similar effects that were observed in adults aged 60 years and older. In the phase IIb trial (NCT05921903), two vaccine doses in immunocompromised adults elicited immune responses similar to one dose in healthy adults aged 50 years old and above with an acceptable safety profile. It is likely that future RSV vaccine recommendations will include those for younger adults in high-risk groups.

## Monoclonal antibodies

Until recently, the only prophylaxis against severe RSV disease was a short-acting mAb, palivizumab. However, since late 2022, a second longer-acting mAb, nirsevimab, has been licensed and subsequently introduced in countries across the globe, with further longer-acting mAbs in the late stages of development.

Palivizumab is a mAb providing passive immunity against RSV disease. It is produced using recombinant DNA techniques in a mammalian cell line. It targets an epitope in the A antigenic site of the RSV F protein responsible for fusing the virus and host cell, thus inhibiting the virus from entering the host cell [93]. Palivizumab was first licensed in the 1990s for prophylactic use in high-risk infants to prevent RSV-associated hospitalizations. Palivizumab has a short half-life of 18–21 days and as such is given as monthly injections during the RSV season to those infants deemed high risk. It is very expensive but is safe and moderately effective in reducing RSV hospitalization rates and serious complications in very high-risk infants [94]. A systematic review of palivizumab for preventing severe RSV infection in children found that it reduced RSV-related hospitalization by 56% but had little impact on mortality [63]. Interestingly, palivizumab was associated with fewer wheezing episodes after a year, highlighting the importance of preventing severe RSV to improve long-term lung health.

Nirsevimab is a new long-acting recombinant mAb providing passive immunity against RSV. Produced in a mammalian cell line, it is directed against the Ø antigenic site of the F prefusion protein of RSV to inhibit the membrane fusion required for viral entry, thus neutralizing the virus. Nirsevimab has an extended half-life (65–70 days), giving protection for at least 5 months based on both clinical and pharmacokinetic data [95]. Nirsevimab was licensed in the UK in November 2022 to prevent RSV-LRTI in infants during their first RSV season. It is safe and effective in reducing MA RSV-LRTI and RSV hospitalization, in both term-born and pre-term infants [96,97]. Nirsevimab efficacy against hospitalization for RSV-LRTI was 83.2% (95% CI: 67.8–92.0), and 75.7% in very severe RSV-LRTI (95% CI, 32.8–92) [98]. Nirsevimab is suitable for high-risk children born preterm and/or with serious heart or lung disease [99]. In Galicia, Spain, a universal nirsevimab immunization programme was introduced to all infants born between September 2023 and March 2024. Early data has shown excellent uptake of immunization with 91.7% coverage, and RSV-LRTI hospitalizations were reduced by 89.8% (IQR 87.5–90.3) for infants immunized with nirsevimab. The number needed to immunize to avoid one RSV-LRTI hospitalization was 25 (IQR 24–32) [100]. Whilst nirsevimab has been approved by the MHRA since November 2022, it is not in general use within the UK. The Joint Committee on Vaccination and Immunisation in the UK recommends implementing a continuous, year-round approach for passive or maternal immunization programmes instead. This strategy is preferred to ensure vaccination coverage and for reasons of operational effectiveness [101].

There is currently one product that is nearing completion of phase 3 clinical trials for RSV immunizations. It is an extended half-life mAb (clesrovimab) from Merck/MSD targeting the RSV F protein. In October 2024, MSD/Merck released topline data from their phase 3 placebo-controlled phase 2b/3 trial evaluating a single dose of clesrovimab administered to healthy preterm and full-term infants (birth to 1 year of age). The primary efficacy endpoint of the trial, the reduction in incidence of MA RSV-LRTI requiring ≥1 indicator of LRTI or severity compared with placebo through 5 months post-dose, was 60.4% (95 % CI: 44.1, 71.9, *P*<0.001). Clesrovimab also reduced RSV-associated hospitalizations by 84.2% (95 % CI: 66.6, 92.6, *P*<0.001) and RSV-LRTI hospitalizations by 90.9% (95 % CI: 76.2, 96.5). Clesrovimab reduced the incidence of severe MA RSV-LRTI by 91.7% (95 % CI: 62.9, 98.1) [102].

Suptavumab, a mAb targeting site V, was more effective than palivizumab at neutralizing RSV in preclinical studies. However, during phase 3 clinical trials, isolates of RSV-B were found to have 2 aa substitutions that resulted in loss of suptavumab-neutralizing activity. It was not known at what frequency these substitutions would naturally occur, or if they were more frequent in the suptavumab group [103]. This highlights the need for post-licensure surveillance for nirsevimab and other licensed mAbs.

Two other long-acting mAbs are in clinical development. An investigational mAb (TNM001) by Trinomab Biotechnology is currently being investigated in a phase 2 trial (NCT05630573) comparing TNM001 with placebo in healthy term and preterm infants. The other candidate (RSM01) has completed a phase 1 trial (NCT05118386). It is an investigational mAb under development by the Bill and Melinda Gates Medical Research Institute aiming to provide an affordable mAb to LMICs. It has an extended half-life (79.1 days) formulation that targets the antigenic site Ø of the F protein. Thus far, there are no concerns regarding the safety profile and bioavailability [104].

## Therapeutic treatments

Currently, treatment options for RSV disease are extremely limited, and the primary focus is on supportive care. There are a small number of treatment options already in use, with limited to modest therapeutic effects, as well as a number of treatments in development. A selection of these is outlined below.

### Ribavirin and ziresovir

Ribavirin is a purine nucleoside analogue metabolized into triphosphate by cellular kinases. Ribavirin’s phosphorylated derivatives exhibit multiple mechanisms of action, contributing to its broad-spectrum antiviral properties. Ribavirin is clinically utilized to treat infections caused by hepatitis E virus, hepatitis C virus and influenza. Ribavirin treatment increases the mutation rate of RSV, which is associated with decreased RSV virus production [105]. Ribavirin is also the only antiviral available for the treatment of RSV-LRTI. It is licensed for bronchiolitis via the nebulized route for children 1–23 months old and for life-threatening RSV in immunocompromised children, administered on expert advice via the intravenous route [106]. Ribavirin is not routinely recommended for the treatment of RSV bronchiolitis due to the high cost, uncertainty about its effectiveness and side effect profile. Ribavirin can cause adverse side effects including bronchospasm, pain, rash and conjunctivitis. It is also teratogenic and contra-indicated in pregnancy [107]. A 2007 systematic review assessing the efficacy of aerosolized ribavirin for infants and children with RSV-LRTI showed that the cumulative results of small studies suggested that ribavirin may reduce the number of days hospitalized and the duration of mechanical ventilation. Additionally, the use of ribavirin may be associated with reduced incidence of long-term wheeze following RSV disease. However, most trials lacked sufficient power to provide reliable estimates of the effects [108].

Ziresovir (AK0529, ArkBio) is another oral RSV fusion protein inhibitor produced by Ark Biopharmaceuticals, designed as a treatment for children 1–24 months old, hospitalized with RSV infection. Cytopathic and plaque reduction assays have demonstrated antiviral activity at nanomolar concentrations against RSV-A and RSV-B isolates collected in different regions during different infection seasons [109]. Their phase 3 trial showed a 30% reduction in signs and symptom scores in children admitted to the hospital with RSV disease compared with placebo [110]. The effect was most pronounced in those less than 6 months old and at higher risk of severe illness. Moreover, it demonstrated a significant reduction in paediatric intensive care unit) stay compared with placebo (median 3 vs 8 days, respectively, *P*=0.05) [111]. The viral load was also significantly reduced in the group that received zirevosir [111]. The company is planning to submit an application for licensure [110].

### Treatments in development

Table 2 highlights the products that are currently in development or under investigation for the treatment of patients with RSV infection. Therapeutic treatments primarily focus on targeting either the viral proteins or the immune response to infection. Strategies that impact viral growth and replication include an l-protein inhibitor (EDP-323), a viral replication inhibitor (zelicapavir) and a derivative of remdesivir (deuterium hydrobromide). Strategies focusing on the innate immune response to infection include XW001 and GB05.

**Table 2.** Treatments for RSV infection currently in clinical development. Created from ‘not yet recruiting/recruiting/active, not recruiting/(recently) completed’ studies registered on ClinicalTrials.gov (25 October 2024)

Product	Phase	Target	Mechanism	Route	Population/comments
LiveSpo Navax (*Bacillus subtilis* and *Bacillus clausii*)	na	Respiratory microbiome	Impact on respiratory microbiome	Nasal spray	Infants with RSV and bacterial co-infection
Wharton’s Jelly umbilical cord mesenchymal stromal cells (ProTrans^®^)	1	‘Tissue resident and bloodborne immune cell’	‘Anti-inflammatory and anti-apoptotic’	Intravenous	Hospitalized adults with RSV/SARS-CoV-2/influenza/HMPV
ALVR106	1/2	Polyclonal CD4/8 virus-specific T-cells	Multi-virus specific T-cell therapy	Intravenous	Adults with HSCT and RSV/influenza/HMPV/PIV Plans for ongoing development
XW001	1b/2a	Interferon pathway	‘based on human interferon lambda 1’	Inhaled	Children with RSV
EDP-323	2	L protein inhibitor	Inhibits viral replication	Oral	Healthy adults challenged with RSV Plans for ongoing development
Obeldesivir (ODV; GS-5245)	2	Nucleoside inhibitor (remdesivir derivative)	Inhibits viral replication	Oral	Non-hospitalized adults
Ribavirin, molnupiravir and favipiravir	2	Varied	Varied	Oral	Adults (outpatients)
Zelicapavir (EDP-938)	2b	Non-fusion replication inhibitor	Modulates the viral N protein	Oral	Non-hospitalized adults with RSV
Deuterium hydrobromide (VV116)	2/3	Nucleoside inhibitor (remdesivir derivative)	Inhibits viral replication	Oral	Infants with RSV
Interferon α1b (GB05)	3	Interferon pathway	‘To supplement endogenous Interferon α1b’	Nebulized	Children<2 y with RSV infection
Azithromycin	3	MMP-9 pathway	MMP-9 pathway	Oral	Children with severe RSV infection

Azithromycin is a macrolide antibiotic that promotes IFN-β production in virus-infected bronchial epithelial cells from asthmatic and COPD donors and shows *in vitro* anti-viral activity against rhinoviruses [112,114]. Azithromycin has not been related to anti-viral activity in models of RSV infection in mice [115]. However, *in vitro* and *in vivo* studies have suggested that azithromycin has anti-inflammatory effects by decreasing the levels of airway matrix metalloproteinase (MMP)-9 during RSV bronchiolitis, attenuating acute and chronic airway inflammation [115]. The secondary analysis of the Azithromycin to Prevent Wheezing following severe RSV bronchiolitis-II (APW-RSV) clinical trial (NCT02911935) demonstrated that azithromycin therapy in children hospitalized with RSV bronchiolitis had a short-term anti-inflammatory effect in reducing upper airway MMP-9 levels. However, the reduction in MMP-9 levels did not relate to subsequent RW post-RSV [116].

A 2023 meta-analysis analysing the efficacy of azithromycin in acute bronchiolitis and wheezing in children under 2 years found moderate-quality evidence that azithromycin may reduce hospitalization duration. However, low-certainty evidence suggested that it did not reduce the need for paediatric intensive care treatment and did not prevent further wheezing episodes [117]. The use of antibiotics to treat viral disease should be treated with caution. The overuse of antibiotics has significantly contributed to the rise in antimicrobial resistance. If azithromycin proved effective at reducing the severity of RSV-LRTI, confirmation of a bacterial coinfection may be required prior to administration.

Ongoing research into the development of RSV therapeutics is still an area of high interest. Human T-cells have been considered a target for future therapeutic agents against RSV. At present, the novel RSV vaccines developed have all been primarily designed to elicit neutralizing antibodies. However, recent mouse model studies suggest that human T-cells play a key role in controlling RSV infection. Specifically, primed human CD8+ T-cells or CD4+ T-cells effectively and independently control replication of RSV in human lung tissue in the absence of an RSV-specific antibody response. ALVR106 is an allogeneic, polyclonal T-cell therapy against Flu, HMPV, PIV and RSV infections. ALVR106 is produced through a process where peripheral blood mononuclear cells are first exposed to specific peptide mixtures. Subsequently, these cells undergo ex vivo expansion with activating cytokines, resulting in Th1-polarized and polyfunctional T-cells, able to kill viral target cells. In the phase 1 first-in-human clinical trial, ALVR106 did not show alloreactivity and was shown to be safe for use in haematopoietic cell transplant or solid organ transplant patients [118]. This supports the development of RSV vaccines, which also elicit T-cell responses to improve RSV vaccine efficacy [119]. However, T-cell-based therapy research is on early clinical assessment.

## Concluding remarks

**Fig. 2.**
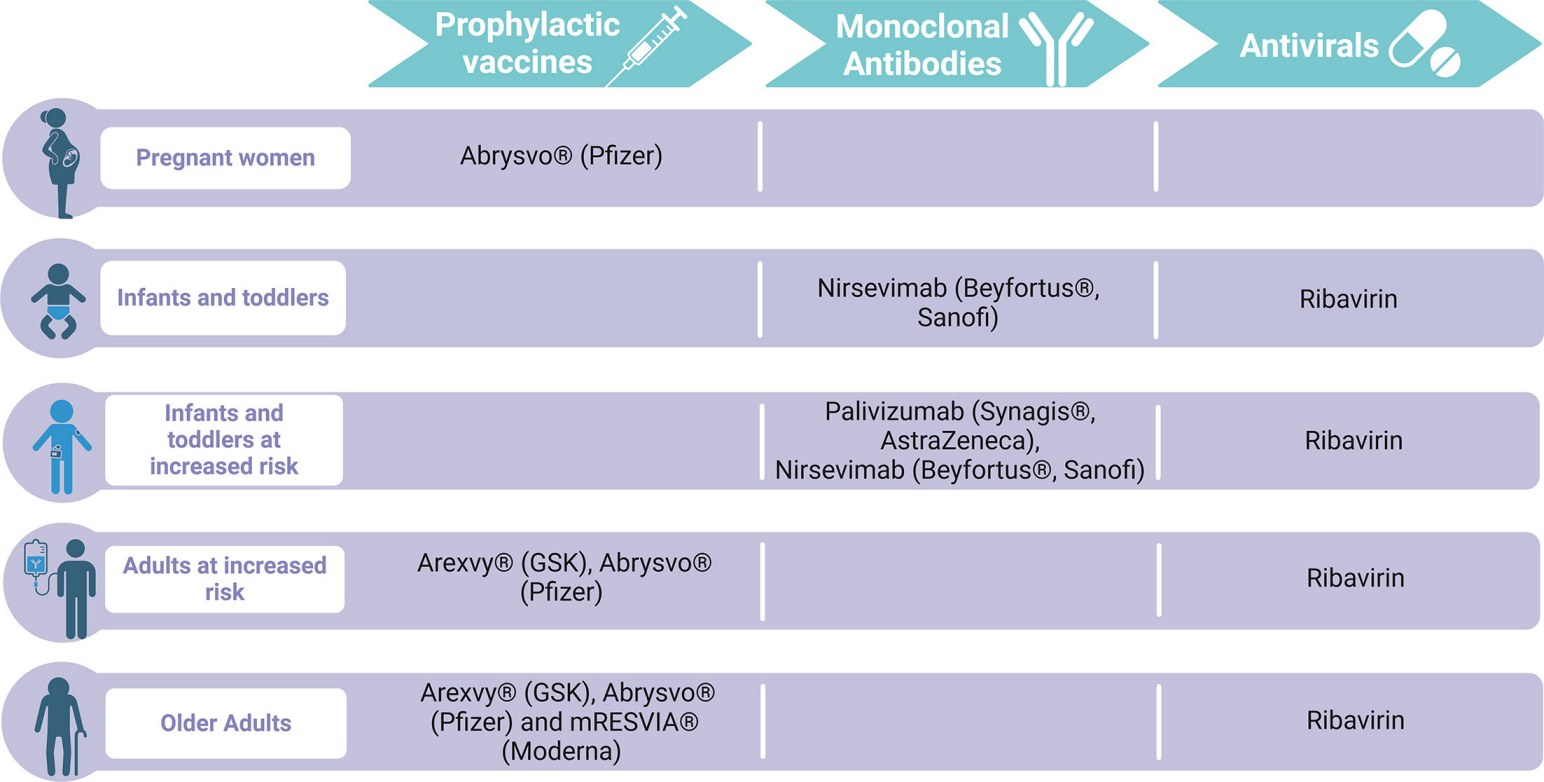
Prophylactic and therapeutic measures authorized for RSV infection.

Nearly 70 years after the discovery of RSV, we finally have licensed vaccines and effective immunoprophylactics (summarized in Fig. 2). It is likely that these significant advancements in RSV research will be a turning point in the morbidity and mortality attributed to RSV. There are vulnerable populations that are not currently covered by the licensed vaccines. Infants and children over the age of 6 months are no longer protected by the maternal vaccine administered during pregnancy. The need for a paediatric vaccine, of which several are in development, is paramount.

RSV has circulated for decades with little evolutionary pressure. The introduction of effective vaccines and widely used immunoprophylactics such as nirsevimab may result in mutations that render these methods of disease prevention less effective. Post-licensure surveillance and sequencing programmes will be critical to monitor circulating strains of RSV to identify the emergence of mutations that may result in viruses that are resistant to vaccines or mAbs. We need to be equipped to respond to changes in vaccine efficacy with easily modifiable vaccine platforms.

Inequality in vaccine distribution and rising vaccine hesitancy will also pose a barrier to effective RSV prevention. Ensuring a global cost-effective supply of vaccines and treatments now needs to be the priority of policy makers and health agencies.

## Abbreviations

ARD: acute respiratory disease
COPD: chronic obstructive pulmonary disease
EMA: European Medicines Agency
ERD: enhanced respiratory disease
EU: European Union
FI: formalin-inactivated
GBS: Guillain–Barré syndrome
GMFR: geometric mean fold rise
GMTs: geometric mean litres
HMPV: human metapneumovirus
LAVs: live-attenuated vaccines
LEEI: low-energy electron irradiation
LMICs: low- and middle-income countries
LNPs: lipid NPs
LRTI: lower respiratory tract infection
MA RSV-LRTI: medically attended RSV-associated LRTI
MATISSE: Maternal Immunization Study for Safety and Efficacy
MHRA: Medicines and Healthcare Products Regulatory Agency
MMP: matrix metalloproteinase
MVA: modified vaccinia Ankara
NP: nanoparticle
NS2: non-structural protein 2
post-F: postfusion F
pre-F: prefusion F
RENOIR: RSV Efficacy Study in Older Adults Immunized against RSV Disease
RSV: respiratory syncytial virus
RW: recurrent wheeze
SIIV: seasonal inactivated influenza vaccine
US FDA: United States Food and Drug Administration
VLPs: virus-like particles
